# Infarcted Warthin tumor with mucoepidermoid carcinoma-like metaplasia: a case report and review of the literature

**DOI:** 10.1186/s13256-018-1941-3

**Published:** 2019-01-14

**Authors:** Kenji Yorita, Hideyuki Nakagawa, Katsushi Miyazaki, Junya Fukuda, Satoshi Ito, Makoto Kosai

**Affiliations:** 1 Department of Diagnostic Pathology, Japanese Red Cross Kochi Hospital, 2-13-51, Shinhonmachi, Kochi-city, Kochi 780-8562 Japan; 2 Department of Otorhinolaryngology, Japanese Red Cross Kochi Hospital, 2-13-51, Shinhonmachi, Kochi-city, Kochi 780-8562 Japan; 3 Department of Otorhinolaryngology, Institute of Medical Biosciences, Tokushima University Graduate School, 2-50-1, Kuramoto-city, Tokushima 770-8503 Japan; 4 Department of Radiology, Japanese Red Cross Kochi Hospital, 2-13-51, Shinhonmachi, Kochi-city, Kochi 780-8562 Japan; 5 Kosai’s Clinic for Otorhinolaryngology, 2-169-1, Hitotsubashi-town, Kochi-city, Kochi 780-0981 Japan

**Keywords:** Warthin tumor, Metaplasia, Mucoepidermoid carcinoma, Necrotizing sialometaplasia

## Abstract

**Background:**

Warthin tumor is a common, benign, painless salivary gland neoplasm. Rarely, Warthin tumors show large areas of squamous metaplasia; such Warthin tumors are called metaplastic or infarcted Warthin tumors because they are occasionally accompanied with tumor necrosis. The histological distinction between mucoepidermoid carcinomas and the metaplastic portions of Warthin tumors can be challenging; without a genetic study, mucoepidermoid carcinomas can be misdiagnosed as metaplastic Warthin tumors. We report a case of infarcted Warthin tumor partly showing mucoepidermoid carcinoma-like epithelial metaplasia. Only two cases of infarcted Warthin tumor similar to our case have been reported.

**Case presentation:**

A 69-year-old Japanese man presented with a right parotid tumor. He had noticed the swelling on his right buccal region 1 year previously; the lesion had rapidly enlarged, with associated pain, 1 month previously. A radiological examination revealed a mass in the tail of the right parotid gland. Superficial parotidectomy was performed. On histological examination, the mass showed typical focal features of Warthin tumor; other areas showed coagulation necrosis of the tumor. These areas were surrounded by non-oncocytic epithelium comprising squamous and mucinous epithelial cells. Although cellular atypia of the non-oncocytic epithelium was not observed, a mixture of squamous and mucinous cells and lack of abundant lymphoid tissue mimicked low-grade mucoepidermoid carcinoma. Based on the results of fluorescence *in situ* hybridization, *MAML2* gene rearrangement was not present in the typical portions of Warthin tumor and the mucoepidermoid carcinoma-like lesion. Therefore, a metaplastic or infarcted Warthin tumor was diagnosed. Our patient was disease-free 8 months after surgery.

**Conclusions:**

Clinicians need to know that pain is a clinical symptom of infarcted/metaplastic Warthin tumor. Pathologists should be aware that a metaplastic Warthin tumor can mimic a low-grade mucoepidermoid carcinoma. Our case showed a mucoepidermoid carcinoma-like lesion that was confined near the area of tumor necrosis, and neither cytological atypia nor apparent invasive growth was present. These findings appeared to be histological clues of a metaplastic Warthin tumor rather than a mucoepidermoid carcinoma. Careful clinicopathological evaluation as well as genetic studies are needed to clarify the distinction between mucoepidermoid carcinoma and metaplastic portions of Warthin tumors.

## Background

Warthin tumor (WT) is a common, benign, salivary gland neoplasm; it consists of a bilayered oncocytic epithelium lining a ductal, papillary, and cystic arrangement in an abundant lymphoid stroma. WTs mainly occur in the parotid gland and affect males in their sixth to seventh decade of life [1]. WTs can show squamous metaplasia of the tumor epithelium; Seifert *et al.* have reported metaplastic WTs with large areas of squamous metaplasia [2]. Metaplastic WTs are rare, and their incidence ranges from 0% (0/278 cases) [3] to 7.6% (21/275 cases) [2]. Metaplastic WTs can also be called infarcted WTs because they are occasionally accompanied by extensive necrosis, fibrosis, and inflammation [4]. Metaplastic WTs may develop mucinous epithelial metaplasia [5, 6], which can pathologically mimic a low-grade mucoepidermoid carcinoma (MEC). WTs rarely coexist with malignant epithelial neoplasms, such as the squamous cell carcinoma (the most common [7]) and MEC; 27 cases of MEC in or coexisting with WT have been described [7–21]. Pathological differentiation of a metaplastic WT from a MEC can be difficult because some metaplastic WTs have been reclassified as MECs based on clinicopathological and genetic studies [17, 22]. Thus, the use of a pathological diagnosis only has limitations in distinguishing metaplastic WTs from MECs. Most MECs are genetically characterized by a t(11;19)(q21;p13) translocation and a *CRTC1*-*MAML2* gene fusion or a t(11;15)(q21;q26) translocation and a *CRTC3*-*MAML2* gene fusion [23]. Typical WTs and metaplastic WTs are considered to have no translocations seen in MECs [22, 24–26]; however, a few opposite results have been previously reported [27, 28]. Fluorescence *in situ* hybridization (FISH) to detect *MAML2* gene rearrangement appears to serve as one of the useful tools to clarify whether the metaplastic portions of WTs are different from the MECs or whether they could be the origin of MECs. The present case is of an infarcted WT with MEC-like metaplasia, and it appears to be the third reported case [5, 6]. We performed a pathological and genetic evaluation to make the distinction between tumor metaplasia and MEC.

## Case presentation

A 69-year-old Japanese man (height, 158 cm; weight, 72 kg; body mass index, 28.8 kg/m^2^) was referred to our hospital because a right parotid gland tumor had rapidly enlarged and developed spontaneous pain 1 month previously. He had noticed the swelling on the buccal region 1 year previously. He had a medical history of hypertension and type 2 diabetes mellitus; he had also undergone surgical resection for gastric lipoma (15 years ago) and urothelial carcinoma (7 years ago). He was receiving oral medication for hypertension and type 2 diabetes mellitus. Medical follow-up revealed no recurrence of urothelial carcinoma. He was living with his wife and had been smoking cigarettes for 30 years, but quit 9 years ago. He had consumed one beer per week for over 40 years. His family and environmental history were unremarkable, and his employment history was not available. At admission, his blood pressure was 164/86 mmHg, but his other vital signs were normal: temperature, 36.4 °C; pulse, 80/minute; respiratory rate, 12/minute with O_2_ saturation of 100% at room air. The physical and neurological examinations were unremarkable except for tenderness in the region of his right parotid gland. The results of complete blood count, serological test, and dipstick urine test were within normal limits.

**Fig. 1:**
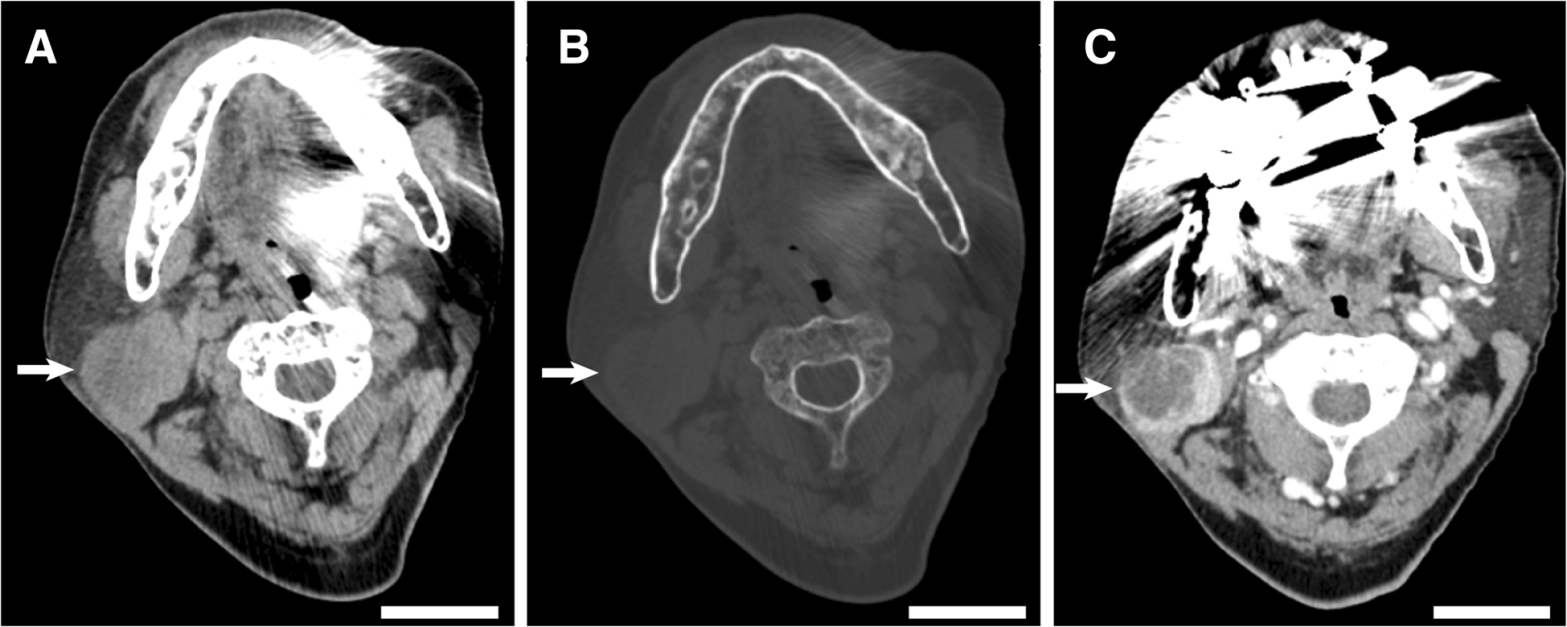
Radiographic images of the tumor. **a**–**b** Axial non-enhanced computed tomography images in soft tissue window (**a**) and bone window (**b**) show a nodular lesion (arrow) in the right parotid gland. **c** Contrast-enhanced computed tomography image shows the cystic and solid appearance of the tumor (arrow). *Bars* represent 5 cm

**Fig. 2:**
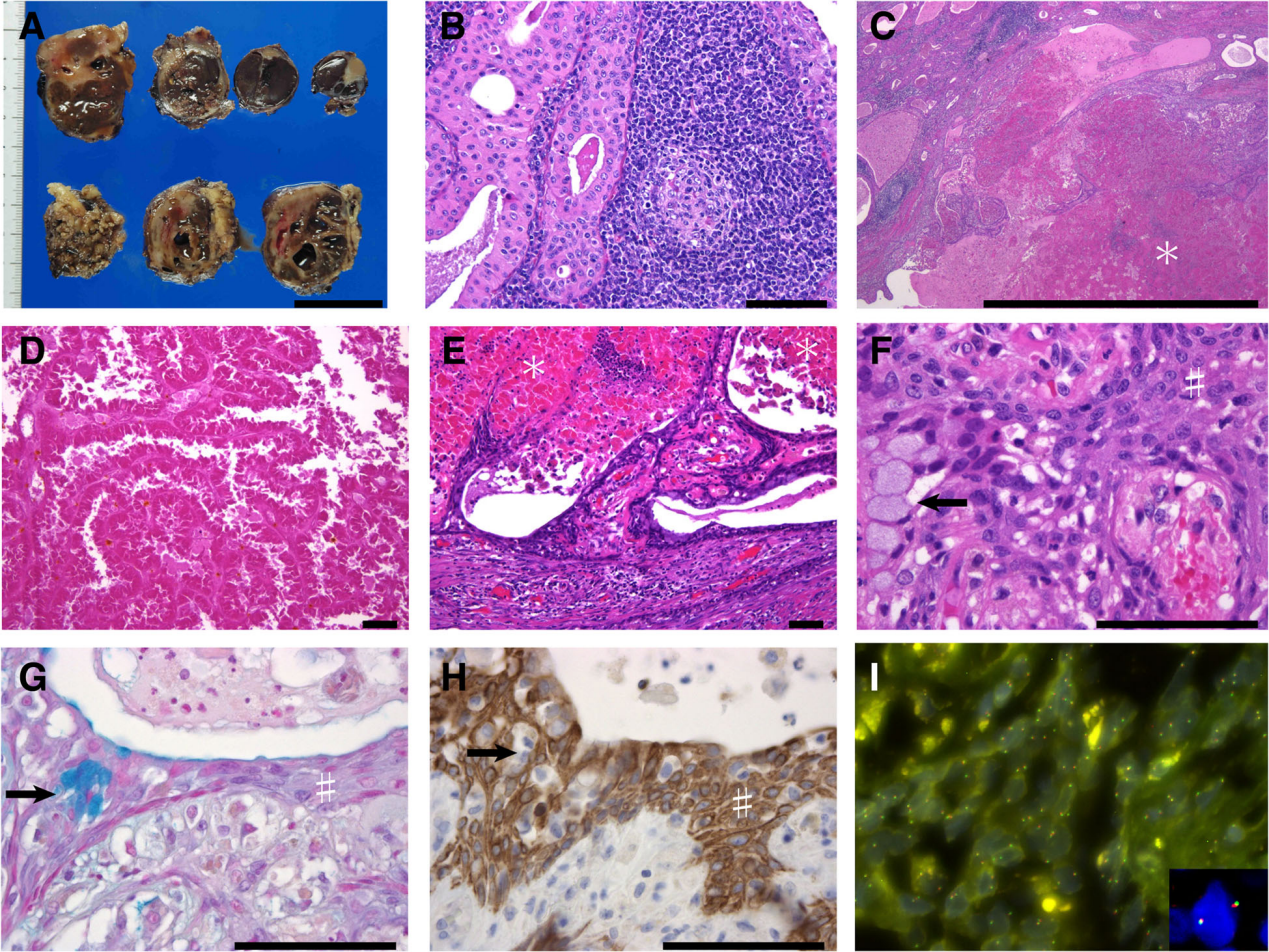
Macroscopic and microscopic findings. **a** The macroscopic appearance of the cut surfaces of the tumor fixed in formalin. Solid and cystic appearance is shown. **b**–**f** Microscopic appearance of hematoxylin-and-eosin stained sections of the tumor. The tumor contains typical features of Warthin tumor (**b**). The tumor shows focal areas of eosinophilic change (**c**–**e**, indicated by * in **c** and **e**), which suggests coagulation necrosis of the tumor. The periphery of the necrotic area consisted of a non-oncocytic epithelium comprising mucinous and squamous cells (**e**, **f**, low and high magnification). Alcian blue staining reveals mucinous cells (**g**). The squamous cells are diffusely positive for cytokeratin 5, and mucinous cells are negative for this marker (**h**). In **f**, **g**, and **h**, mucinous cells are indicated by an *arrow* and squamous cells are indicated by #. **i** Fluorescence *in situ* hybridization does not show *MAML2* gene rearrangements in the metaplastic component. An inset in **i** shows a nucleus without *MAML2* rearrangement. *Bars* in **a** and **c** represent 3 cm; bars in **b**, **d**–**h** represent 100 μm

A computed tomographic examination showed a mass of 5-cm diameter located in the superficial lobe of his right parotid gland (Fig. 1a–b), and the mass had solid and cystic components based on contrast imaging (Fig. 1c). Serum levels of squamous cell carcinoma antigen and soluble interleukin-2 receptor were within reference limits. WT was clinically suspected based on the location in the tail of the right parotid gland, cystic morphology, gender, and age; however, a malignant salivary gland tumor could not be excluded. Superficial parotidectomy was performed for diagnosis and treatment. On gross examination, the formalin-fixed mass was solid, and the cut surface of the tumor had a grayish appearance (Fig. 2a). No fluid content was observed. A whole-mount preparation of the mass was performed. On histological examination, the mass showed typical focal features of WT, that is tubulocystic growth of bilayered, columnar, and oncocytic epithelium associated with abundant lymphoid stroma (Fig. 2b). In the other portion of the tumor, approximately 60% of it, there were eosinophilic materials suggesting coagulation necrosis of the tumor; the materials were surrounded by a non-oncocytic epithelium comprising non-keratinizing squamous cells and mucinous cells (Fig. 2c–h). The non-oncocytic epithelium was associated with a fibrous stroma or granulation tissue, but not with abundant lymphoid stroma (Fig. 2e). Granulomatous inflammation involving foreign body-type giant cells was also seen. The non-oncocytic epithelium showed neither distinct cellular atypia nor apparent invasive growth, but the fibrosis adjacent to the non-oncocytic epithelium showed a desmoplastic reaction. Thus, low-grade MEC could not be excluded. On immunohistochemical examination, the squamoid cells in the MEC-like lesions were positive for cytokeratin 5 (CK5) and p63, and mucinous cells were negative for these markers (Fig. 2h). The necrotic materials were diffusely positive for epithelial markers (AE1/AE3 and cytokeratin 7) and negative for CK5 and p63. The Ki-67 positive ratios in the WT and MEC-like components were similar; both components’ ratios were less than 5%. No diffuse immunoreactivity of p53 was observed. Results of FISH showed *MAML2* gene rearrangements were not present in the typical portions of WT and the MEC-like lesion (Fig. 2i). Therefore, we diagnosed this tumor as a metaplastic or infarcted WT. Our patient was discharged without sequelae and was disease-free 8 months after the surgery.

## Discussion

We have described a 69-year-old Japanese man with a WT showing squamous and mucinous metaplasia and large areas of tumor necrosis. Our case was probably similar to a case of infarcted WT described by Eveson and Cawson [4]. The patient’s age and gender in our case were typical for WT; however, the symptom was unusual because a slowly growing painless mass is typical for WT. The tumor in the present case increased in size and developed pain within a short time, which mimicked a malignant tumor. Infarcted WTs tend to be more associated with pain than WTs are; it has been reported that pain occurs in 7% of WTs and 25% of infarcted WTs [4]. A clinician should be aware that an aggressive clinical course can be encountered in a case of infarcted WT.

Metaplastic change and infarction in WT appears to be closely related because squamous metaplasia has been observed in 35% of 20 cases of infarcted WT [4], and infarcted WTs have been observed in 70% of 21 cases of WTs with large areas of squamous metaplasia [2]. Causes of metaplasia in WTs include ischemia and/or infarction, irradiation, and biopsy [2, 4–6, 29]. In our case, biopsy and irradiation had not been performed; infarction was possible although the cause of the infarction remained unknown. A similar phenomenon, called necrotizing sialometaplasia, has also been observed in normal salivary gland tissue; ischemic salivary gland tissue can develop squamous and mucinous metaplasia. Thus, it appears that similar to normal salivary gland tissues, WTs can develop metaplasia due to ischemia.

WTs showing both squamous and mucinous metaplasia may be rare because Rotellini *et al.* reported that the incidence of such cases was 0.2% among cases of WTs (1/444 cases) [28]. However, Rotellini *et al.* [28] selected metaplastic WTs that had significant areas of metaplasia, and Seifert *et al.* reported that WTs with focal, not diffuse, areas of both squamous and mucinous metaplasia were observed in 22% of WTs (48/217 cases) [2]. Thus, WTs requiring the exclusion of a diagnosis of a MEC may be not be infrequent; however, published cases similar to our case are rare. Only two cases of infarcted WT with MEC-like metaplasia have been reported, but they lacked a genetic study and a sufficient description of the histological differentiation between MEC and MEC-like metaplasia [5, 6]. Infarcted WTs can show cytological atypia of the metaplastic epithelium and accompanying fibrosis and inflammatory infiltrate [4–6], and the metaplastic portions can mimic the desmoplastic tumor reaction as was seen in our case. In the present case, the MEC-like lesion was confined adjacent to the tumor necrosis; such metaplasia was not observed in the tumor away from the necrosis. WTs may be more likely when MEC-like lesions are confined near the necrotic areas in the WTs and the MEC-lesions do not form mass lesions. Further case studies are needed to identify useful histological clues to differentiate MEC-like WT from MEC.

However, pathological diagnosis of a metaplastic WT can be challenging. Ishibashi *et al.* showed that five cases of WT showing diffuse metaplasia that were extracted from among cases of WT were reclassified as cases of MEC (Warthin-like MEC) because all these cases were positive for *CRTC1-MAML2* gene fusion; the cases comprised mainly female patients and lacked the typical bilayered oncocytic epithelium typical of WT [22]. Thus, a low-grade MEC associated with abundant tumor-associated lymphoid stroma can be misdiagnosed as a metaplastic WT. Ishibashi *et al.* also reported that a Warthin-like MEC and a metaplastic WT are difficult to distinguish, and the most reliable finding in distinguishing WT from Warthin-like MEC is the presence of bilayered oncocytic epithelium [22]. In addition, the metaplasia of WT may show a precursor or early phase of MEC. In 27 previously reported cases of MEC in or coexisting with WT [7–21],14 cases were accompanied by squamous or mucinous metaplasia, [8, 11–14, 16, 19], and six of the 14 cases were confirmed or suspected to have MECs that had transformed from the metaplastic portions of WT [11, 16, 19]. Rotellini *et al.* reported that two of eight cases of metaplastic WT showed a *MAML2* gene rearrangement in the squamous cells, but not in the mucinous cells and oncocytic cells [28]. However, 28 cases of metaplastic WT did not show *MAML2* gene rearrangement [22, 25, 26]. Thus, the squamous metaplasia in WTs does not appear to typically show *MAML2* gene rearrangement. Careful clinicopathological evaluation as well as genetic studies are needed to confirm whether the metaplastic portion of a WT can be the origin or early phase of a MEC.

## Conclusion

We have reported a case of infarcted or metaplastic WT that required an exclusion of low-grade MEC at the metaplastic portion. The MEC-like component lacked significant cytological atypia, apparent invasive growth, or *MAML2* gene rearrangement as determined by FISH. In addition, our patient’s age and gender were typical for WT. Based on these findings, it was concluded that the present tumor was WT. Pathologists need to be aware that MEC-like changes can occur in infarcted or metaplastic WT.

## Abbreviations

CK5: Cytokeratin 5
FISH: Fluorescence *in situ* hybridization
MEC: Mucoepidermoid carcinoma
WT: Warthin tumor
